# Both sides now: evolutionary traits of antigens and B cells in tolerance and activation

**DOI:** 10.3389/fimmu.2024.1456220

**Published:** 2024-08-09

**Authors:** Youngjae Hong, Kihyuck Kwak

**Affiliations:** ^1^ Department of Microbiology and Immunology, College of Medicine, Yonsei University, Seoul, Republic of Korea; ^2^ Brain Korea 21 PLUS Project for Medical Science, College of Medicine, Yonsei University, Seoul, Republic of Korea

**Keywords:** B cell activation, B cell tolerance, BCR Signaling, antigen format, vaccine, autoimmunity

## Abstract

B cells are the cornerstone of our body’s defense system, producing precise antibodies and safeguarding immunological memory for future protection against pathogens. While we have a thorough understanding of how naïve B cells differentiate into plasma or memory B cells, the early B cell response to various antigens—whether self or foreign—remains a thrilling and evolving area of study. Advances in imaging have illuminated the molecular intricacies of B cell receptor (BCR) signaling, yet the dynamic nature of B cell activation continues to reveal new insights based on the nature of antigen exposure. This review explores the evolutionary journey of B cells as they adapt to the unique challenges presented by pathogens. We begin by examining the specific traits of antigens that influence their pathogenic potential, then shift our focus to the distinct characteristics of B cells that counteract these threats. From foundational discoveries to the latest cutting-edge research, we investigate how B cells are effectively activated and distinguish between self and non-self antigens, ensuring a balanced immune response that defends against pathogenic diseases but not self-antigens.

## 1 Introduction

The life of a B cell is not much different from ours. As we go through years of education and evaluation, B cells, from birth to death, are also evaluated for their ability to initiate appropriate BCR signaling (1). Beginning in the bone marrow, immature B cells undergo central tolerance mechanisms to ensure their appropriate recognition of non-self antigens. Following their migration to secondary lymphoid organs, mature B cells that retain self-reactivity are subject to peripheral tolerance mechanisms, often resulting in the induction of anergy or deletion (2). Here, the concept of tolerance primarily pertains to the induction of unresponsiveness in B cells towards self-antigens, ultimately serving to prevent the emergence of autoimmune diseases stemming from autoreactive B cells.

Despite these safeguards, the journey of B cells is far from over. For a fully mature naïve follicular B cell, breaking tolerance represents a significant challenge. Transitioning into antibody-secreting plasma cells requires the breaking of tolerance through classical antigen-induced B cell receptor (BCR) signaling along with co-receptors. Once activated, these stimulated B cells differentiate into germinal center (GC) B cells for extensive educational programs (3). Within GCs, a process known as somatic hypermutation occurs, selecting GC B cells with high-affinity mutations for differentiation into plasma cells, while directing relatively low-affinity clones to become memory B cells for future encounters with pathogens (4). Proper maintenance and breakdown of immune tolerance are crucial for preventing the development of autoimmune diseases while simultaneously ensuring a broad spectrum of immune repertoire.

Early studies have covered the general scheme of B cell development, demonstrating the strategies employed by the immune system to remove the autoreactive clones by the central tolerance mechanism. Random V(D)J recombination may give rise to autoreactive clones which are given a chance for redemption by receptor editing, a RAG-mediated gene rearrangement process. If the clone remains self-reactive, clonal deletion removes the autoreactive repertoire (2, 5). However, central tolerance is not sufficient to entirely remove autoreactivity. From here, the peripheral tolerance mechanism plays the key role by inducing anergy to silence the autoreactive clones. Furthermore, antigen stimulated B cells are required to meet additional checkpoints, most notably, T cell help. This ‘second signal’ is essential for the survival of activated B cells as those that fail to receive T cell help eventually undergo activation-induced cell death (6). As described briefly, B cells have layers of tolerance mechanisms and these traditional, yet solid, concepts of B cell tolerance have guided the study of B cells to great progression. However, the key question of self and non-self recognition mechanisms, particularly by B cells, remains unanswered.

The adaptive immune system has evolved to discriminate against self and non-self antigens in a way to boost or hinder the immune response toward foreign or self-antigens, respectively. Through the regulation of B cell tolerance, B cells that should be activated are turned on, while those that should remain inactive are rendered inactive. This review dissects the mechanism behind early B cell activation into two concepts: 1) the characteristics of self and pathogen-derived antigens and 2) the intrinsic design of B cells to discriminate between self and non-self. These two distinct aspects are closely intertwined, yet more to be uncovered. Ideally, an advanced understanding of the B cell tolerance mechanism will provide new insights into the field of autoimmune diseases as well as vaccine development.

## 2 Nature of the antigen

The discovery of the interaction between pathogen-associated molecular patterns (PAMPs) of microorganisms and the pattern recognition receptors (PRRs) of innate immune cells serves as a paradigmatic illustration of the pathogen alert system (7). Similarly, our body has defense mechanisms in different levels of the immune system to protect ourselves from a diverse array of pathogens. Recent work on B cell tolerance reveals mechanisms governing how B cells discern and respond to self and non-self antigens (8, 9). As it is important for B cells to recognize and effectively respond to pathogens, many groups have directed their efforts towards the early phase of B cell activation, specifically immediate-early BCR signaling.

All B cells have a set level of activation threshold. Different subsets of B cells harbor intrinsic strategies to manage the activation threshold. For example, studies on the nature of GC B cells have revealed the qualitative difference in BCR signaling, highlighting the increased antigen-affinity threshold compared to that of naive B cells (3). This is due to the intrinsic re-wiring of the BCR signalosome, notably the recruitment of active phosphatases to the BCR complex (10). On the other hand, the naive population seems to rely on the antigen format to somehow lower or boost the activation strength in the means of BCR signaling. The resulting magnitude of BCR signaling, when surpassed the activation threshold, may drive the B cell towards activation and terminal differentiation or, when maintained below the activation threshold, keep the B cell unresponsive (4). The following chapters review the impact of antigens on BCR signal transduction and, in reverse, the distinct features of B cells that respond to antigen-mediated activation.

### 2.1 Membrane vs. soluble antigens

Physiologically, B cells encounter cognate antigens in their membrane form as follicular dendritic cells, dendritic cells, and macrophages deliver the antigens, in their native forms, to the secondary lymphoid organs (11). Also, pathogens such as microorganisms or viruses often present repeated arrangements of pathogenic particles on their membranes (12). However, circulating self-antigens remain uncaptured by antigen-presenting cells (APCs), keeping their soluble form. Therefore, in order to maintain simplicity based on a binary perspective, one might readily conclude that membrane-bound antigens are likely pathogens, whereas soluble antigens are endogenous. Early studies have reported the effectiveness of membrane-bound antigens in eliciting a strong B cell response (5, 13). Unraveling the molecular mechanism became the next step for B cell biologists.

**Figure 1:**
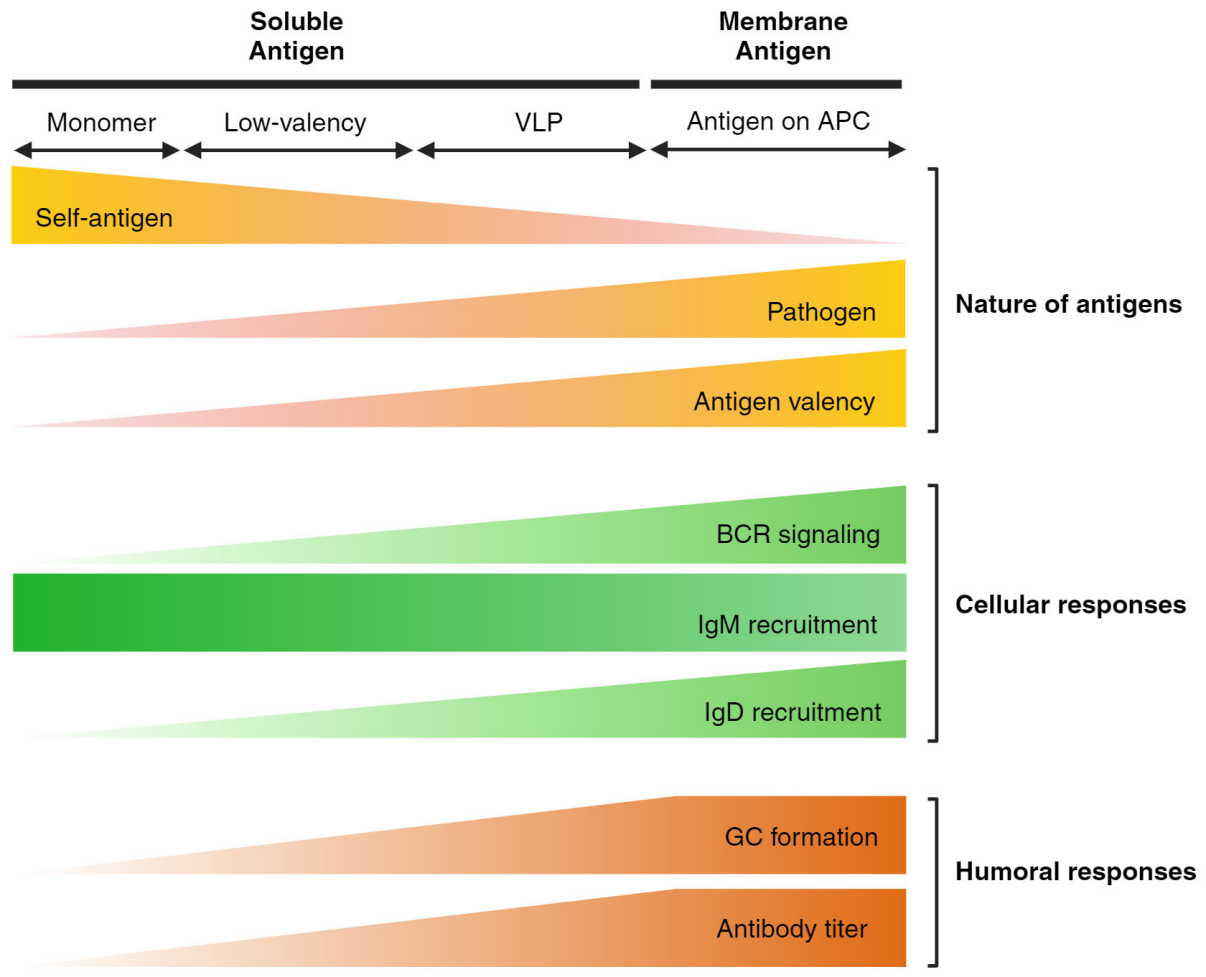
Features of antigens and B cell responses on different antigen formats. The schematic illustrates the impact of antigen formatting on initial B cell responses and the overall humoral response. Self-antigens, typically low-valency, are ignored by the self-reactive B cell repertoire through the downregulation of IgM, leading to anergy. However, IgD is retained as a pathogen alert system, specifically recognizing multivalent antigens in both soluble and membrane-bound forms. Consequently, it is well-established that multivalent VLP structures or antigens presented by APCs through membrane-bound recognition elicit a stronger humoral response.

While not entirely within the primary scope, anergic mouse model studies demonstrate the potency of membrane-bound antigens compared to soluble antigens. Anergic B cells can be artificially induced by meeting two criteria: 1) bearing self-antigen specific BCRs and 2) constant exposure of that particular antigen. The well-established anergic model incorporates cross-breeding of the HEL-specific BCR expressing mouse line (MD4) and the soluble HEL (sHEL) secreting mouse line (ML5) (14). However, when MD4 line is crossed with the membrane-bound HEL (mHEL) presenting mouse line (KLK3), significant defects are found during the accumulation of mature B cells in the secondary lymphoid organs (13). The anergic population arises only when moderate, tonic BCR signaling is induced by soluble self-antigens. In the case of membrane HEL presentation, excessive BCR signaling removes the autoreactive B cells by clonal deletion. This highlights the distinct response of B cells to membrane-bound versus soluble antigens and underscores the importance of antigen presentation format in shaping B cell tolerance and activation. In a similar aspect, this chapter focuses on the recognition of antigen formatting, specifically the membrane-bound and soluble forms, by fully mature, naive follicular B cells (**Figure 1**).

The qualitative advantage of membrane-bound antigens over soluble antigens has been described for decades. Similarly, BCR signaling induced by soluble, multivalent antigens are stronger than mono- or di-valent stimuli, *in vitro* (15–18). Recently, these ‘particulate’ antigens have been studied in depth demonstrating the membrane-bound antigen-like characteristics of such antigens (19, 20). However, as the size of the particulate antigens vary from a few nanometers to cell-size micrometer beads, particulate antigen studies fail to clearly define the antigen format; in fact, particulate antigens are viewed as an intermediate between the two (this review, in a strictly dichotomic manner, will consider particulate antigens as soluble, multivalent antigens). So far, a direct comparison of both qualitative and quantitative strength of BCR signaling in response to membrane-bound and soluble antigens has not been described. Hence, the following section primarily focuses on the molecular events that occur during membrane-antigen interaction and the possible advantages conferred to B cells in this context.

#### 2.1.1 Experimental tools

Key characteristics of B cells were first found by, literally, ‘looking at’ them. For example, traditional approaches could not capture the spreading characteristics of B cells, which is discussed in the next section. These initial imaging-based works became the groundwork for B cell biologists and robust efforts were made incorporating imaging techniques.

The BCR signalosome represents a highly dynamic compartment incorporating the Igα/Igβ-coupled BCR molecules and the intracellular signaling and adaptor molecules. Although the well-established model of BCR signaling suggests the series of phosphorylation cascades in a sequential manner, this perspective may underrate the complexity of the actual event. Thus, traditional proteomic analysis based on western blotting or flow cytometry is not sophisticated enough to grasp such intricacy. Efforts were made by B cell biologists to overcome these experimental limitations. To strictly focus on the spatiotemporal interaction between the BCR signaling molecules, B cell biologists have turned to microscopy as the foremost tool, enabling visualization of this intricate process to, at least, some extent. Total Internal Reflection Fluorescence Microscopy (TIRFM) is especially valuable for the study of BCR signaling as it can visualize not only the surface molecules but also the intracellular signaling molecules that are recruited to the signalosome with high resolution. In fact, TIRFM can penetrate to a depth of approximately 100nm which is in the range of recruited early BCR signaling molecules (21). Recently, super-resolution imaging has become the go-to technique as Stochastic Optical Reconstruction Microscopy (STORM) imaging can overcome the diffraction limits of 200nm, which is substantially larger than the actual size of a single molecule. These technologies afford scientists the nanoscale organization of the BCR signalosome. Additionally, the imaging-based techniques incorporated in B cell studies are well described in the following review.

In the following studies discussed below, the membrane-bound form of antigen was mimicked, *in vitro*, by affixing the antigen onto planar lipid bilayers (PLB) or plasma membrane sheet (PMS) placed on a glass coverslip. This tool establishes an effective membrane-bound antigen format which can be directly used for TIRF and/or STORM imaging as B cells are able to spread and contract on the antigen-bearing glass.

#### 2.1.2 Formation of immune synapse and BCR microclusters

IS formation was first suggested by a theoretical paper by Norcross in 1984 (22). The term ‘synapse’ was borrowed from the nervous system as Norcross hypothesized that T cell interaction with antigen-loaded APCs would mimic the synaptic architecture found in neurons. Almost a decade later, Monks and colleagues first visualized the IS in T cells and additionally discovered the supramolecular activation cluster (SMAC) where molecular interactions occur during cell-to-cell contact (23). In 1999, Dustin and colleagues mimicked the IS formation, *in vitro*, incorporating the planar membrane system proposed by McConnell (24).

A few years later, IS formation in B cells was first discovered by Batista and colleagues by using the PLB system described above (25–28). Similar to the TCR-MHCII architecture, the interaction between membrane-bound antigens and BCRs gives rise to the immune synapse. During this two-phase process, the B cell membrane spreads over the antigens and eventually contracts back to its original form, ‘eating off’ the antigens (28). Remarkably, this entire process occurs within a matter of minutes. BCR signaling serves as a necessary condition for B cell spreading, a phenomenon validated by studies utilizing signaling-deficient BCR B cells (29). Simultaneously, BCRs form microclusters encompassing the membrane-bound antigens. Less is known about *how* BCRs behave so, but several reports have revealed the *why*, highlighting the signaling advantages of such architecture.

These seemingly closely related structures have not been studied in relation to each other. It is widely appreciated that the extent of both IS and BCR microcluster formation, in terms of kinetics and intensity, is dependent on antigen concentration and affinity (28, 29). However, unlike B cell spreading, the initial formation of does not require functional BCR signaling as CD19KO and LynKO murine B cells were still able to form microclusters following membrane-bound antigen engagement (29, 30). Due to these discrepancies, we cannot definitively conclude whether the two phenomena are necessary conditions for each other, two separate mechanisms induced by membrane-bound antigen interaction, or something in between.

Yet, the *why* has been clearly described with convincing evidence in line with the well-established role of IS during TCR-MHCII interaction. During the initial phase of cell spreading, BCR-antigen microclusters form at the periphery of the contact area while integrin-ligand (LFA-1/VLA-4 - ICAM-1/VCAM-1) interactions confer cell-to-cell adhesion at the peripheral supramolecular activation cluster (pSMAC) (25, 26). During the contraction phase, BCR-antigen microclusters migrate to and form the central supramolecular activation cluster (cSMAC) where antigen extraction and internalization takes place (28). The current perspective of these events is that they serve a single purpose: to reduce the activation threshold. In a macro view, antigen recognition, gathering, and extraction at the immune synapse allows B cell activation even at low antigen concentrations which is physiologically more relevant. In a micro view, BCR microclusters have been discussed to amplify early BCR signaling events. Taken together, both concepts support the notion that the immune synapse itself acts as a leverage for enhanced B cell response, especially in scenarios characterized by low antigen concentrations. Thus far, imaging experiments often use anti-IgM antibodies for membrane stimulation and/or IgM-only expressing B cell lines, largely ignoring the role of IgD. As recent findings highlight the distinct distribution of IgM and IgD-associated molecules forming the IgM and IgD protein islands, IgD involvement is a topic too crucial to be disregarded.

In contrast to membrane-bound antigen studies, soluble antigens have gained less interest. PLB system allows researchers to visualize the early BCR signalosome, but only in the membrane-antigen context. A few confocal imaging-based reports have illustrated the general behavior of B cells to soluble stimuli in which BCRs are polarized to form the ‘BCR cap’ (31). However, this is considered a ‘dead signal’ zone which contrasts from the concept of BCR microcluster. This concept originates from studies on membrane-bound antigens, where active BCR signaling is initiated at the periphery but diminishes as BCR microclusters migrate to the cSMAC for antigen extraction (32). In this respect, BCR capping may resemble the dead signal zone, at least visually. Yet, this lacks evidence and the tendency of B cells to form polarized ‘caps’ in response to soluble stimuli requires further investigation.

It has been nearly two decades since the discovery of the B cell immune synapse, a milestone concept that continues to represent a cornerstone in the field of B cell biology. Building upon IS formation, recent studies have focused on the molecular details underlying the crosstalk between signaling molecules, owing to the significant advancements in imaging-based methodologies. Additionally, researchers have made innovative approaches to introduce novel concepts and techniques into B cell research, including but not limited to liquid-liquid phase separation, proximity labeling techniques, and investigations into the role of mechanosensitive channels during cell spreading (33–36).

#### 2.1.3 Mechanosensitive channels

As described above, cell spreading involves active recruitment of the actin and microtubule filaments as well as receptor-ligand mediated adhesion between the membranes (32). Recent studies have focused on the mechanosensitive forces that may result from B cell spreading. Kwak and colleagues touched on the *in vitro* mechanisms of a mechanosensitive ion channel, Piezo1, that may play a crucial role in B cell activation, especially in the membrane-bound antigen format (37). Piezo1 induced enhanced calcium signaling when stimulated by membrane-bound antigens, but not soluble antigens. Furthermore, B cell spreading area was significantly reduced when Piezo1 was knocked-down (KD), reinforcing the impact of Piezo1 in BCR signaling. The physiological role of Piezo1 in B cells remains widely unknown, and further *in vivo* studies may be required to highlight the significance of Piezo1 in eliciting the overall humoral response. Similarly, the role of TRPV2, another mechanosensitive ion channel, in B cell activation was demonstrated by Liu and colleagues (38). Each and every stage of B cell-mediated humoral response, spanning from the formation of the immunological synapse (IS) to the signaling components of B cell receptor (BCR) and ultimately to the secretion of antibodies, was diminished in TRPV2-KO mice. Interestingly, the expression level of TRPV2 correlated with the disease progression of patients with systemic lupus erythematosus (SLE). Recently, Treanor and colleagues investigated the role of TRPV5, another member of the TRPV family, in early BCR signaling (39). Due to its low expression level, TRPV5 showed minimal effects on B cell development and function. However, TRPV5 was recruited to the signalosome in a signaling-dependent manner, partly suggesting a potential role in BCR signaling. Altogether, these studies reveal that the BCR signalosome is more complex than previously understood, particularly in the context of membrane-bound antigens.

This novel mechanism underscores previous findings that antigen rigidity and stiffness, in both soluble and membrane antigen context, are crucial determinants of BCR signaling strength. Tolar and colleagues used atomic force microscopy (AFM) to elucidate the antigen extraction mechanism, demonstrating that it operates in a mechanical force-dependent manner driven by myosin IIa contractility. Highlighting the significance of BCR microclusters, their research demonstrated that the force generated by a single BCR-antigen bond is insufficient to trigger antigen extraction. This finding aligns with previous studies emphasizing the necessity of BCR microclusters for effective signaling (40, 41). Liu and colleagues further evaluated the strength of BCR signaling by varying the concentration of bisacrylamide gels to manipulate the stiffness of the antigen-bound substrate, quantified by Young’s modulus (42). BCR recruitment, the subsequent pSYK signaling, and finally the expression level of CD69, an activation marker of late-stage BCR signaling, were enhanced in antigen substrates with higher stiffness. Similarly, soluble antigens tethered to a rigid substrate induced greater calcium influx than the responses induced by antigens attached to the flexible ssDNA or PEG linkers (20).

Altogether, the results imply that B cells may have the ability to sense micro-tensions, either extrinsically or intrinsically. Thus, the concept of mechanosensitive channels may be applicable to the nature of antigens also: not only macrotensions induced by IS formation but microtensions led by antigen stiffness or increasing antigen valency may be sufficient to aid the initiation of BCR signaling in the means of tension-mediated ion influx.

### 2.2 Antigen arrangement

Nonetheless, B cells, unlike T cells, have the distinct ability to recognize an antigen in its MHC-unloaded native form. Also, certain pathogens may evade the primary defense mechanism (local APCs) of our body and encounter the cognate B cells in their soluble form (43, 44). Consequently, it is conceivable that B cells have evolved mechanisms to augment their response to soluble antigens by recognizing their particular format. As we discussed about the molecular mechanisms behind membrane-bound antigen-mediated BCR signaling, this section transitions to an exploration of how B cells respond to soluble antigens with varying nature.

#### 2.2.1 Antigen spacing

Regularly spaced repeating units of epitopes are recognized as danger signals in our immune system. In fact, Bjorkman and colleagues have suggested a possible mechanism that HIV has evolved, downregulating the density of envelope spike proteins, to perhaps evade the danger signal-mediated clearance (45). In the field of vaccinology, antigen spacing has been a topic of interest for robust antibody response (46–48). Yet, initial B cell activation in response to different antigen spacing remains unclear. To this end, comprehending the molecular models of B cell receptor (BCR) activation is critical to fully understanding the importance of antigen spacing.

The activation model of BCRs has been a topic of controversy for decades, well discussed in previous reviews (49, 50). Two conflicting theories of BCR activation, conformation-induced oligomerization model and the dissociation activation model, have different views in the initial arrangement of IgM and IgD BCRs (49). The latter argues that BCR molecules, initially clustered together, become more dispersed upon engaging with antigens, while the former claims that individual BCR monomers form oligomers upon binding to antigens. However, they both agree on the fact that BCRs are spaced approximately 10~20 nm apart from each other in their antigen-bound form (51, 52). Hence, researchers have engaged in investigating the optimal antigen spacing of antigens for B cell activation.

A conventional approach involved the use of NIP-coupled peptides, facilitating the in-house construction of antigens with the desired number of epitopes or epitope spacing. NIP-peptides with distances of 0, 3, 7, and 24 amino acids were evaluated, yet no discernible functional differences in terms of calcium influx were observed (16). This lack of distinction may be attributed to the fact that a spacing of 24 amino acids corresponds to a mere 8 nanometers apart, a distance that may even shorten if the peptide adopts an alpha-helix structure.

A more sophisticated strategy leveraging DNA origami nanoparticle technology was subsequently employed, wherein antigen-dimer rods (80nm in size) with various distances were examined (20). Among spacings of 7, 14, 21, 28, and 80 nanometers, a spacing of 28 nanometers was identified as the most effective for eliciting maximal calcium signaling. However, it is important to note that this data alone is insufficient to conclusively determine that the most efficient antigen spacing is 28 nanometers. Indeed, several hypotheses can be proposed, as soluble antigens may associate with the BCR in unexpected manners. For instance, it was unexpectedly observed that an 80 nanometer spacing was efficient in inducing calcium influx. One possible explanation for this phenomenon could be that two molecules of 80 nanometer-spaced antigen-dimer rods bound to a single BCR, thereby altering the experimental condition from an 80 nanometer-spaced antigen to a different configuration. The steric hindrance posed by the 80 nanometer-sized rod likely precludes such possibilities from occurring in other antigen-dimer rods.

Similarly, Högberg and colleagues utilized the DNA origami technology to construct an antigen platform with defined distances (53). Taking a biochemical approach, the study focused on the interaction between purified in-house produced antibodies, rather than B cells, and the differentially spaced bi-valent antigens. Interestingly, IgM showed exceptional spatial tolerance for the bi-valent antigens. The opposite could be suggested for IgD: low spatial tolerance may render the molecule unresponsive to low-valency antigens. In this regard, the preference of IgD for multivalent antigens may be explained by the spatial tolerance concept. However, as the study primarily focuses on the binding affinity between antibodies and antigens, the intrinsic B cell behavior following antigen engagement is partly ignored. Thus, it is risky to make a solid conclusion in the context of B cell activation.

#### 2.2.2 Antigen valency

Antigen valency is a prominent topic in both B cell biology and vaccinology. Crotty and colleagues have highlighted the remarkable impact of multivalent antigen (eOD-GT 60-mer) immunization in stimulating a robust humoral response (54). Following this initial report, numerous groups, targeting various virus types, have documented an enhanced antibody response when using multivalent vaccine platforms (46, 55–57). However, it is worth noting that the enhanced humoral response observed may stem from various advantages conferred by multivalent antigens, including improved antigen delivery kinetics, enhanced antigen presentation efficiency, and augmented recruitment of follicular helper T cells to the germinal center (58). All of these factors may be interconnected. Consequently, the enhanced humoral response may be influenced by factors beyond B cell behavior, thus potentially yielding biased results. In order to explore the intrinsic response of B cells to antigen valency, researchers have developed diverse antigen platforms.

Some of the early studies on B cell activation briefly addressed the magnitude of BCR signaling induced by varying antigen valencies (15–18). However, these studies primarily focus on determining the minimal antigen valency required for BCR signaling, thereby supporting one of the BCR activation models. Kiessling and colleagues utilized ring-opening metathesis polymerization (ROMP) to construct multivalent antigens with diverse valencies (e.g., 10, 25, 50, 100, 250, and 500-mer). Antigen-specific A20 B cell line stimulated with higher antigen valencies resulted in stronger BCR signaling, quantified by the amount calcium influx (15). However, as Ubelhart and colleagues highlighted the differential role of IgD in sensing multivalent antigens, studies incorporating cell lines bearing only IgM may not be sufficient to fully grasp the nature of primary naive B cells (59). This may explain why recent work by Bathe and colleagues failed to demonstrate clear valency-dependent calcium influx in IgM-bearing Ramos B cells. In their study, the calcium signaling capacity saturated at relatively low valencies, with the 4-mer antigen showing similar phenotypes to the 60-mer antigen (20).

Schamel and colleagues investigated the role of antigen valency using the NIP-coupled peptide platform, as described previously (16). In this study, primary splenic B cells expressing both IgM and IgD were utilized, although antigen valency was only tested up to 3-mers. Unexpectedly, only minor changes in BCR signaling were observed when stimulated with different antigen valencies. This could potentially be attributed to inefficient antigen spacing, as NIP molecules were arranged only 3 amino acids apart from each other.

Recently, Zikherman and colleagues reported a possible mechanism behind multivalent antigen-led BCR signaling amplification incorporating a more sophisticated platform. Here, they utilized a viral-sized ( ≈ 100 nm) liposome structure displaying desired number of HEL molecules, mimicking the bona fide viruses, along with the HEL-specific MD4 primary B cells (19). Note that antigen-specific B cells may not fully represent the responses of wild-type naive B cells, as those with defined BCR sequences fail to receive any BCR signaling during development, potentially resulting in a different signaling program. Nevertheless, according to their study, multivalent antigens evade the Lyn-mediated signaling negative-feedback loop. It is reasonable to speculate that B cells evolutionarily established a defense mechanism against pathogens bearing multivalent epitopes, such as viruses. This also connects to the concept of self and non-self as multivalent antigens may resemble foreign pathogens with repeated epitopes. To that end, differential B cell response to multivalent antigens is a valuable topic of interest. Like PAMP recognizing PRRs, B cells may form a specialized BCR signalosome compartment in response to multivalent antigens.

The vaccine industry has already embraced the concept of antigen valency, often implemented through virus-like particles (VLPs) (60–62). Nonetheless, studies on multivalent antigen-induced BCR signaling are still in their infancy. For instance, unlike membrane-bound multivalent stimuli, soluble multivalent antigens do not provide a proper environment for IS formation, yet B cells still respond robustly, inducing strong BCR signaling. Unraveling such mechanisms would serve as a bridge between B cell immunology and vaccinology, potentially leading to innovative approaches in vaccine development.

## 3 Nature of the B cell

### 3.1 Surface organization of the BCR signalosome

The previous chapter discussed the initiation of BCR signaling in the antigen aspect. We now transition to the strategies adopted by B cells such as regulation of surface protein level, formation of an efficient signalosome, distinct signaling patterns to diverse stimuli, and differential responses by the twin molecules IgM and IgD, all of which are intricately connected to the nature of the antigens.

#### 3.1.1 Organization of surface receptors

B cells express IgM, but not IgD, in a wide range of spectrum. The varying level of IgM expression is known to be inversely proportional to the cell’s autoreactivity (8). Batista and colleagues used a bead-based counting assay to determine the number of BCR molecules present on primary B cells (63). Among the approximately 320,000 BCR molecules expressed on the B cell membrane, an average of 286,000 are IgD, while about 40,000 are IgM. However, these numbers do not precisely represent BCR expression levels, particularly for IgM, as IgM expression can vary from 19,000 to 83,000 molecules due to the presence of self-reactive populations. This skewed expression of BCR molecules highlights the importance of studying IgD. Current imaging studies exhibit one or more of the following limitations: 1) using PLBs with only anti-IgM stimulus, 2) pre-labeling BCRs with only anti-IgM antibodies, or 3) using non-primary cell lines that express only IgM, as no cell lines carry endogenous IgD. The most optimal activation condition would involve using an antigen with its cognate primary B cell. However, this approach raises another dilemma as manipulating primary B cells raises other technical challenges.

Interestingly, the initial organization of IgM and IgD differs dynamically (63, 64). Both molecules are clustered, but to different extents, in their unactivated native forms. dSTORM analysis of resting primary B cells revealed that IgD molecules tend to exist in a more pre-clustered state, indicated by a Hopkins index of 0.83 compared to IgM’s 0.66. Furthermore, these BCR pre-clusters form distinct protein islands with different co-receptor molecules. Partial evidence suggests that IgM protein island may contain CD45 while IgD protein island couples with CD19, CD81, CD20, and Lyn, in the resting state (65). However, the spatial dynamics rapidly change after antigen interaction. A CTB-Fab-PLA study captured the exchange of lipid compartments, showing the movement of GM1 gangliosides from the IgD region to the IgM region (51), suggesting the possibility of co-receptor exchange during early BCR signaling.

From a subjective perspective, the main issue in B cell studies is the diversity of experimental protocols. The variety of cell types (cell line vs. primary cell), stimuli (anti-BCR antibody vs. cognate antigen), and activation conditions (PLB vs. soluble form) results in a highly diverse array of experimental conditions that can significantly alter results. In fact, BCR signaling is arguably one of the most versatile signaling pathways, highly sensitive to small changes in experimental conditions. Thus, comprehensively understanding the numerous reports from varying contexts remains a challenge, especially at the molecular level. The next step for scientists is to synthesize this diverse information and piece together the puzzle to gain a cohesive understanding of BCR signaling.

#### 3.1.2 Co-receptor engagement

As discussed above, BCRs engage in cross-talk with various co-receptor molecules during BCR signaling. While the roles of individual co-receptors are well-established, they are often understood in a simplistic, binary manner. Initial co-receptor analysis focused on structural studies of the immunoreceptor tyrosine-based activation/inhibition motif (ITAM/ITIM) to determine whether a molecule acts as a positive or negative regulator of BCR signaling. For example, CD19 is well-recognized as a key player especially during membrane-bound antigen stimulation (29). Negative regulators involve CD22, CD32, and CD45, owing to the ITIM motifs they harbor (63, 66–68). When phosphorylated, those ITIM motifs recruit the phosphatases, SHP-1 and SHIP-1 to counter the phosphorylation cascade.

The dynamics of co-receptors have been appreciated by imaging-based studies where CD45 was excluded from both BCR and TCR microclusters during signal transduction (29, 69). Furthermore, recruitment of CD32 to BCR microclusters results in delayed immune synapse formation and, as a consequence, reduced BCR signaling-mediated calcium influx (66, 67). However, the scope of such studies fails to cover the physiological role of the co-receptors, neglecting the interactions between bona fide pathogens and cognate B cells.

Experimental limitations also play a key role in developing a rather dichotomous view of these receptors. For example, the role of co-receptors was often studied by co-ligating the BCR with the target of interest using antibodies specific for the BCR and the target molecule on a PLB system. Such methodology may fail to demonstrate the active cross-talk between surface molecules under physiological conditions as co-receptor may not necessarily engage with the BCR complex but may have distinct roles elsewhere. Nonetheless, these studies provide valuable pieces of evidence that aid in hypothesizing the mechanism behind BCR signaling.

In fact, reports on dual players such as CD45 or Lyn have revealed both positive and negative roles for these molecules at different time points of BCR signaling (68, 70, 71). Yakura and colleagues highlighted the role of CD45 in dephosphorylating Src-family kinases, particularly Lyn, as a negative feedback mechanism (71). However, evidence has also suggested a positive role for CD45, where it dephosphorylates the negative regulatory site of Lyn immediately following BCR activation (72). The current view on these dual players often undermines the possibility that such molecules may play both activating and inhibiting roles in different spatial and temporal windows.

More recently, back-to-back studies highlighted the role of a ‘third-party’ molecule galectin-9 in BCR signaling (73, 74). Galectin-9 directly binds to IgM and CD45, reorganizing the BCR-co-receptor interaction and, thus, acting as a physical modulator of antigen-induced BCR signaling in both soluble and membrane-bound antigen contexts. Additionally, another co-receptor CD22 is indirectly recruited to the signalosome, though the exact mechanism remains to be studied. A subsequent study further emphasized the role of galectin-9 in regulating the activation threshold of B cells (75). These findings provide new insights into non-traditional regulatory mechanisms that may be crucial for B cell activation.

Efforts have been made for decades to delve into the intense cross-talk between BCR and co-receptor-signaling molecules. A super-resolution study partially visualized the dynamic movement of CD19, suggesting its migration from the IgD to the IgM compartment upon antigen stimulation (51, 64). Non-imaging-based techniques have recently been employed in B cell studies, effectively reproducing the well-known characteristics of early BCR signaling (36). Yet, the application of such novel technologies requires solid experimental competency along with innovative approaches by researchers.

#### 3.1.3 Distinct roles of IgM and IgD in pathogen recognition

The biggest challenge that still remains long-lasting in the field of B cells is, perhaps, the secret behind IgM and IgD dual expression in naive B cells. During B cell development, immature B cells express only IgM, while fully mature B cells express both IgM and IgD (76). Despite slight differences in the hinge region, both BCRs have identical cytoplasmic and transmembrane domains as they originate from the same primary mRNA strand, generated by alternative splicing (76). However, studies have reported different roles for each BCR during development, initial activation, and differentiation. The concept of dual BCR expression is well-studied in the context of anergic B cells, where differential expression levels of IgM and IgD were first reported (77). Yet, the topic of IgM versus IgD remains unexplored in our context of B cell activation.

Ongoing debates about self-reactive B cells, which constitute about 20% of the total B cell population, have questioned the reason for their existence. The most logical conclusion is that tolerance-mediated clearance of all self-reactive B cell clones might leave the body vulnerable to molecular self-mimicking pathogens. In this regard, Goodnow and colleagues focused on the role of IgD during B cell development to the secondary lymphoid organs. An IgD knockout (KO) study revealed that naive IgM^low^ B cell populations overexpressed CD138 in an unregulated fashion, highlighting the role of IgD as a safeguard in the accumulation of self-reactive clones as mature follicular (Fo) B cells (78). Similarly, Zikherman and colleagues conducted a study on IgM^+/-^ IgD^-/+^ heterozygous mice, which develop B cells expressing either IgM or IgD due to allelic exclusion of lymphocytes (9). IgD-only B cells were predominant among the mature follicular B cells, partially supporting the previous conclusion. The presence of IgD, rather than the absence of IgM, was responsible for this skewed selection, as IgD-only and wild-type (IgM and IgD-expressing) B cells showed equal competition, while IgM-only B cells showed a disadvantage against WT B cells in the mature follicular compartment. However, these *in vivo* studies fail to cover the signaling aspects giving rise to these results. Additionally, the roles of both BCRs during development is a separate topic to be discussed which may differ from our context. Nonetheless, these results provide some insight into the nature of the two BCRs.

Connecting this concept to the context of early B cell activation, maintaining the expression of IgD is logically plausible. Supporting this idea, Uberhart and colleagues reported the unique characteristic of IgD being responsive to multivalent antigens and possibly membrane-bound antigens (59). Interestingly, IgD can bind low-valency antigens but fails to initiate BCR signaling and subsequent BCR internalization. However, Goodnow and colleagues have reported otherwise (78), leaving the nature of IgD to be further studied. For now, it can be said that anergic B cells downregulate IgM expression to tune down their responsiveness to soluble self-antigens but maintain IgD expression to respond to foreign, multivalent, and/or membrane-bound antigens. However, much remains to be understood, particularly regarding IgD, as B cell studies have yet to explore its involvement in various B cell activation conditions.

Recently, cryo-EM technology has provided a new perspective for B cell biologists (79, 80). Analyses of IgM-BCR and IgG-BCR complexes, which include the Iga/b subunits, have primarily focused on the resting state of these complexes. The findings corroborate previously established characteristics of B cells and offer detailed insights into the molecular interactions within these complexes. These details could be crucial for resolving longstanding debates, such as the activation mechanism of BCRs or the differences between IgM and IgD. However, the IgD-BCR complex was not included in the study. Furthermore, current cryo-EM analyses rely on in-house synthesized proteins, which may differ from the native BCRs from naive B cells. Nonetheless, the further application of high-resolution techniques such as cryo-EM in our context would provide valuable insights and potentially clarify the remaining questions about early BCR signaling.

### 3.2 Differential BCR signaling

Finally, this last section covers the most fundamental aspect of B cell activation: the BCR signaling. In general, BCR signaling is carried on by a series of phosphorylation cascades which ultimately turns on and off the key transcription factors such as FoxO, JNK, ERK, NFAT, and NF-kB. ERK, NFAT, and NF-kB are robustly turned on while FoxO is inhibited by activated AKT molecules (81). Overall, these transcription factors modulate cell proliferation, survival, and further differentiation. As just described, traditional understanding of signaling pathways is characterized as linear, top-down, and sequentially ordered series of events. However, in most cases, that is not the case. Likewise, the BCR signalosome is a complex compartment involving dynamic interaction between the signaling molecules. As recent studies suggest that multiple layers of activation threshold may act on B cells for full activation, comprehensive understanding of the nature of BCR signaling may reveal the mechanisms by which the B cells modulate their activation threshold in response to antigen stimulation.

Interestingly, the initiation of BCR signaling may not be sufficient for the full activation of B cells. Zikherman and colleagues have shown the qualitative difference in BCR signaling induced by monomeric sHEL versus multivalent synthetic virus-like structure (SVLS)-HEL antigens (19). Surprisingly, when IgHEL B cells were treated with ‘equipotent’ concentrations of each antigen that induced similar magnitudes of BCR signaling, comparable amounts of phospho-ERK were induced. However, robust NF-kB activation was only observed in response to the multivalent stimulus. This implies a possibility that BCR signaling may have additional thresholds besides the initial activation threshold. One hypothesis may be the impact of calcium as a second messenger, as multivalent antigens have shown their ability to not only induce a strong calcium peak but also sustain the cytoplasmic calcium level for several minutes. In fact, efficient NFAT activation requires sustained cytoplasmic calcium level, which has not yet been fully elucidated in this context (82–84).

These studies support the theory that B cells may harbor a multi-layered tolerance mechanism. A single event, such as antigen encounter, may not be sufficient to fully activate the B cell. A combination of antigen stimulation and T cell interaction is known to strongly induce NF-kB signaling, *in vitro* (19). Similarly, LPS, a widely used mitogen for B cells, stimulates the TLR4-led Myd88/IRAK4 pathway which induces robust NF-kB activation (85). In fact, BCR signaling is known to synergize with TLR signaling to enhance NF-kB pathway to a greater extent, thereby leading to greater activation-induced cytidine deaminase (AID) activity (86). Thus, taken altogether, B cells may harbor multi-layered thresholds which must be overcome to become fully activated. Already well-known pathogen alert systems such as TLR signaling or T-B cell interaction are both sufficient and necessary for full NF-kB functionality. However, less has been reported in the perspective of antigen-led BCR signaling. Recent data predominantly, though not conclusively, suggest that soluble multivalent or membrane-bound antigens may be sufficient to, even without T cell help, break naïve B cells’ tolerance for terminal differentiation, ultimately leading to antibody responses and enhanced protection.

## 4 Conclusion

**Figure 2:**
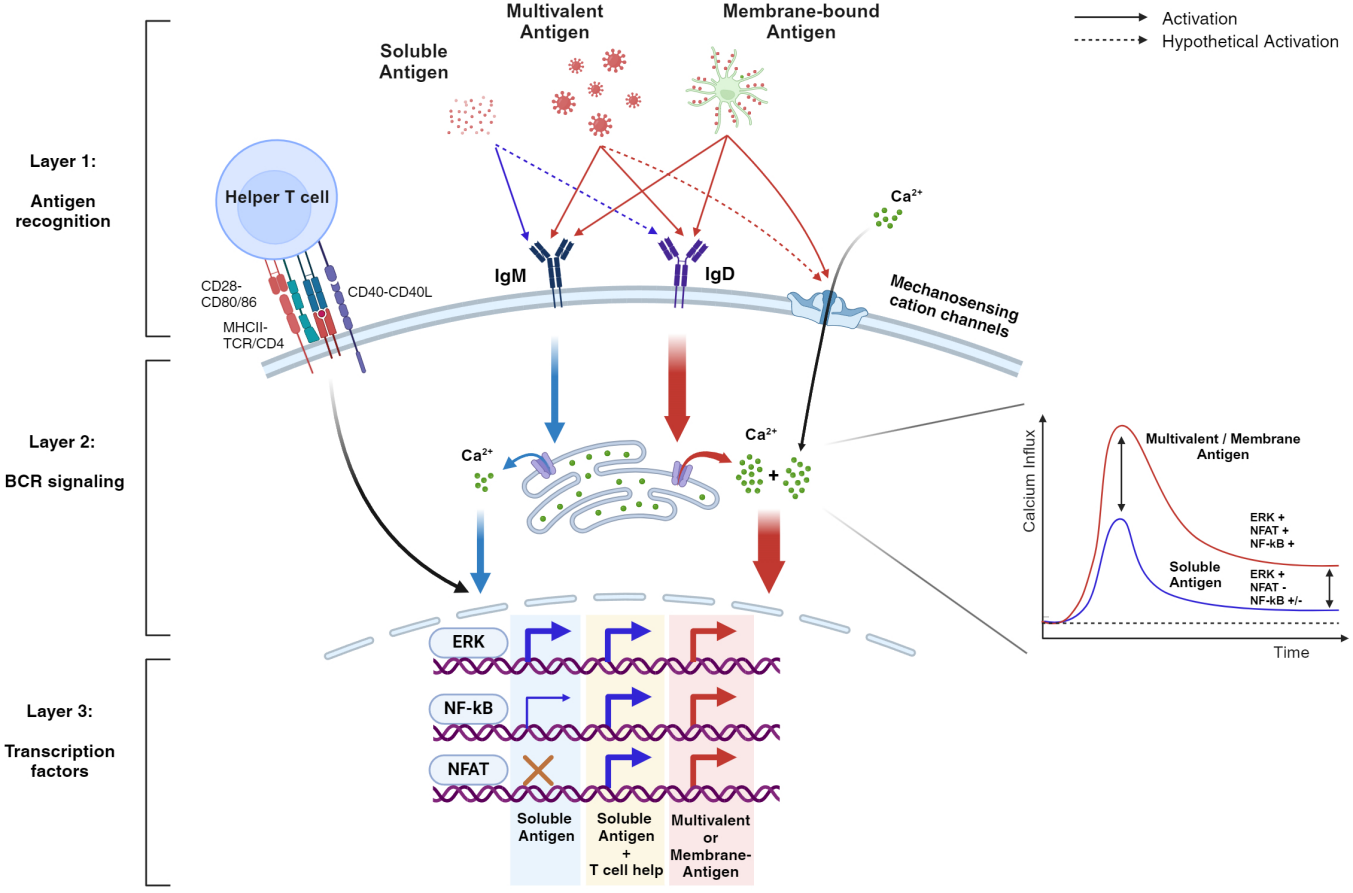
BCR signaling is differentially regulated by various antigen formats qualitatively and quantitatively. This hypothetical schematic demonstrates the layers of B cell tolerance from antigen recognition to intracellular signaling. During antigen recognition, soluble stimuli (blue arrows), unlike multivalent and/or membrane-bound stimuli (red arrows), generally bind to IgM, resulting in weak intracellular signaling that fails to sustain cytoplasmic calcium levels. Consequently, key transcription factors such as NF-kB and NFAT are weakly activated or fail to activate, respectively. However, with T cell help, the weakened signaling is rescued, leading to the activation of these key transcription factors. In contrast, multivalent stimuli can drive B cell activation without T cell help by amplifying early BCR signaling and maintaining high cytoplasmic calcium levels. This is due to their ability to engage IgD in the signalosome and form additional structures such as the immune synapse (not illustrated), which activates mechanosensitive ion channels. As a result, transcription factors are effectively activated, providing robust protection against pathogens. Note that the diagram highlights both quantitative and qualitative signaling differences between soluble and multivalent stimuli. In addition, the dotted arrows represent aspects that are not yet fully understood. Evidence for the activation of various transcription factors comes from multiple studies, indicating that the actual events may vary depending on the activation context.

The traditional view on B cell activation was “on or off”, rather simplifying the B cell behavior. However, recent work illustrates profound details that regulate B cell activation, conferring layers of tolerance mechanisms to prevent unnecessary humoral responses. As a result, B cells can be turned on and off at appropriate contexts. Turning off is well-described by the central and peripheral tolerance mechanisms in which self-reactive B cells are modified in their V(D)J sequence for redemption, completely deleted from the immune repertoire, or silenced in the means of anergy (5, 87). This review focused on how B cells regulate the ‘on switch’ by bringing together the most fundamental findings along with recent studies that not only support the initial concept of B cell activation but further suggest the molecular basis of how B cells overcome tolerance during and after antigen engagement (**Figure 2**).

Although this review is categorized into two chapters—the nature of antigens and B cells—it is difficult to distinctly separate these concepts, as antigen characteristics and B cell behavior are closely interrelated. It is conceivable that B cells have evolutionarily developed mechanisms to remain silent against self-antigens while eliciting maximal responses toward pathogens. This is supported by the heightened B cell response induced by membrane-bound and multivalent antigens. Consequently, multivalent antigens, which resemble viruses or bacterial pathogens with repeated units of foreign proteins, may trigger a novel BCR signaling pathway that enhances the immune response. The specifics of this pathway, however, remain to be investigated.

A possible candidate for the enhanced B cell response could be the involvement of IgD, which has been significantly overlooked due to the experimental limitations of the PLB system. While IgD’s response to low-valency antigens is minimal, it is notably sensitive to multivalent antigens. Therefore, it is reasonable to assume that IgD may play a significant role in responding to membrane-bound antigens. Although the roles of IgM and IgD during B cell development have been examined using IgM- and IgD-only B cell mouse models, these studies fail to address the activatory roles of both BCRs in fully mature, naive B cells. The qualitative signaling differences between IgM and IgD following antigen stimulation remain unclear.

Exploring B cell tolerance not only intrigues many B cell biologists, but also offers crucial clinical insights for autoimmune research and vaccinology. Understanding the pathways or key molecules regulating B cell tolerance would be beneficial for vaccine development, as adjuvants targeting these pathways could be designed to enhance the immune response quantitatively or, ideally, qualitatively. For example, the development of a Piezo1 agonist as a vaccine adjuvant for T cells has been recently reported (88). While such approaches have a long way to go, they could potentially provide a better adjuvant platform compared to current TLR-based stimulation. Conversely, an antagonist could be used to combat autoimmune diseases by reducing the immune response’s magnitude toward self-antigens. Ongoing research on B cell tolerance not only captivates researchers but also promises substantial clinical applications, suggesting transformative advancements in both fundamental science and industrial sectors.
